# Aluminium oxide nanoparticles compromise spatial learning and memory performance in rats

**DOI:** 10.17179/excli2017-1050

**Published:** 2018-02-14

**Authors:** Imen M'rad, Mustapha Jeljeli, Naima Rihane, Pascal Hilber, Mohsen Sakly, Salem Amara

**Affiliations:** 1 Laboratoire de Physiologie Intégrée, Faculté des Sciences de Bizerte, Université de Carthage, Tunisie; 2 Institut Supérieur des Sciences Humaines de Tunis, Université El Manar, Tunisie; 3 Centre de recherche sur les fonctionnements et dysfonctionnements psychologiques CRFDP EA 7475, Université de Rouen Normandie, France; 4 College of Education Afif, Ministry of Education Shaqra University, Kingdom of Saudi Arabia

**Keywords:** aluminium oxide nanoparticle, hippocampus, oxidative response, spatial memory

## Abstract

Recently, the biosafety and potential influences of nanoparticles on central nervous system have received more attention. In the present study, we assessed the effect of aluminium oxide nanoparticles (Al_2_O_3_-NPs) on spatial cognition. Male Wistar rats were intravenously administered Al_2_O_3_-NP suspension (20 mg/kg body weight/day) for four consecutive days, after which they were assessed. The results indicated that Al_2_O_3_-NPs impaired spatial learning and memory ability. An increment in malondialdehyde levels with a concomitant decrease in superoxide dismutase activity confirmed the induction of oxidative stress in the hippocampus. Additionally, our findings showed that exposure to Al_2_O_3_-NPs resulted in decreased acetylcholinesterase activity in the hippocampus. Furthermore, Al_2_O_3_-NPs enhanced aluminium (Al) accumulation and disrupted mineral element homoeostasis in the hippocampus. However, they did not change the morphology of the hippocampus. Our results show a connection among oxidative stress, disruption of mineral element homoeostasis, and Al accumulation in the hippocampus, which leads to spatial memory deficit in rats treated with Al_2_O_3_-NPs.

## Introduction

Nanoparticles (NPs) have several industrial, military, and medical applications, among other uses. In nanotechnology, the devices or engineered materials used have sizes ranging from 1 to 100 nm (Borm et al., 2006[9]; Oberdörster et al., 2005[35]; Sajid et al., 2015[43]). NPs can have negative impacts on the environment and cause many diseases because of their specific properties, which include high surface reactivity, shape, and special structure (Amara et al., 2014[5]; Chen et al., 2008[11]; Oberdörster et al., 2005[35]; Sajid et al., 2015[43]). Cellular uptake and penetration of NPs into the blood and lymph are easy because of the small size of such particles (Chen et al., 2008[11]; Oberdörster et al., 2005[35]). NPs may be deposited in different body organs and tissues; however, their accumulation at body sites can lead to toxicity (Åkerman et al., 2002[4]; Ballou et al., 2004[7]; Borm et al., 2006[9]; De Jong et al., 2008[13]). Furthermore, some studies have shown that NPs can cross the blood brain barrier (BBB) (Chen et al., 2008[11]; Cupaioli et al., 2014[12]). It has been found that NPs can provoke neurotoxicity and alter cognitive functions in mice and rats. In addition, the aforementioned effects were found to be a function of NP type, duration of treatment, and administered dose (Lockman et al., 2004[28]; Sharma and Sharma, 2007[46]; Sharma et al., 2012[47]).

Aluminium oxide nanoparticles (Al_2_O_3_-NPs) are abundantly produced and used in various consumer, medical, domestic, and industrial products (Balasubramanyam et al., 2009[6]; Monterio-Riviere et al., 2010[32]). They also have various military applications (Miziolek, 2002[31]). Aluminium nanoparticles (Al-NPs) are used as fuel in propellants, as they have a high enthalpy of combustion and a pyrotechnic characteristic (Ghanta and Muralidharan, 2013[20]; Miziolek, 2002[31]; Wagner et al., 2007[51]). They are also used to manufacture electrical components and batteries (Piercey and Klapoetke, 2010[38]). It has been proposed that Al-NPs can be used as a carrier system to increase drug solubility (Tyner et al., 2004[50]) .

Previous studies have shown that Al impairs the cholinergic system and causes learning deficits (Abdel-Aal et al., 2011[1]; Kumar et al., 2009[24]). Al can also provoke cell depletion in the cortex and hippocampus (Kumar et al., 2011[25]; Wang et al., 2014[52]). Consequently, it can affect learning and memory ability. However, the mechanism by which Al induces a negative effect on memory is not clearly understood yet. 

Some studies on the toxicity of Al_2_O_3_-NPs have indicated that the particles change spatial cognition capability. In addition, a study conducted on experimental animal brains showed that Al_2_O_3_-NPs may cause oxidative stress by damaging membrane lipids and disturbing the antioxidative enzyme defence system (Karmakar et al., 2014[23]; Prabhakar et al., 2012[40]; Shah et al., 2015[45]). Furthermore, it has been demonstrated in different rodents that Al_2_O_3_-NPs can cause neurotoxicity by inducing cytotoxic effects, genotoxic effects, and inflammatory events in the brain (Chen et al., 2008[11]; Li et al., 2009[27]; Prabhakar et al., 2012[40]; Shrivastava et al., 2014[48]).

There is limited information on the toxicity of Al-NPs to the brain. Therefore, in the present study, we assessed the effect of Al_2_O_3_-NPs on spatial memory. Additionally, we investigated the response profiles of oxidative stress and acetylcholinesterase (AChE) activity in the hippocampus. Furthermore, Al accumulation, mineral element homoeostasis, and the morphology of the hippocampus were also analysed.

## Materials and Methods

### Animals

Twenty-four male Wistar rats (180-220 g), 2-3 months old, were obtained from SIPHAT (Ben Arous, Tunisia) for the study. The animals were acclimatised to the study environment for at least one week before experiments were conducted. The rats were housed in groups (n = 6 per group) in polypropylene cages under standard conditions. The room was well ventilated and maintained at a temperature of 22 ± 4 °C. Additionally, the animals were kept under a 12/12 h light/dark cycle (light on at 09:00 AM and light off at 09:00 PM) and allowed free access to water and food. The protocols used in this work were approved by the Medical Ethical Committee for the Care and Use of Laboratory Animals of Pasteur Institute of Tunis (approval number: LNFP/Pro 152012).

### Drugs

Al_2_O_3_-NPs (Cat No. 544833; Sigma-Aldrich, St. Louis, MO, USA) were used in this study as a dry powder. The product specifications are as follows: gamma phase alumina NPs; particle size, ˂ 50 nm (transmission electron microscopy, TEM); and surface area, 40 m^2^/g (Brunauer-Emmett-Teller). 

### Characterisation of Al_2_O_3_-NPs

#### TEM analysis 

The particle size and shape of the Al_2_O_3_-NPs were determined using a Tecnai G2-200KV system (FEI Company, Hillsboro, OR, USA). Sample preparation for observation by TEM was done as follows. First, the powder was mixed with EtOH, after which ultrasonic dispersed particles were deposited onto a lacey-carbon-coated copper grid. 

#### Powder X-ray diffraction (XRD) analysis

The crystal structure of the NPs was assessed by powder XRD using an advanced X-ray diffractometer (D8 Advance; Bruker Corporation, Billerica, MA, USA) at 40 kV and 30 mA. Scanning was performed with 2.2 kW Cu anode radiation at a wavelength of 1.54 A˚. About 250 mg of Al_2_O_3 _was deposited on the sample holder for scanning over a range of 10-100 °C. 

### Treatment

Al_2_O_3_-NPs were suspended in fresh sterilised physiological saline solution (9 ‰ sodium chloride) at a concentration of 20 mg/ ml. The suspension was sonicated (Vibra-Cell model CV 18; Sonics, Newtown, CT, USA) for 30 min before it was administered (Amara et al., 2014[5]). In order to prevent Al_2_O_3_-NP agglomeration, the temperature of the sonicator was kept below 30 °C. The animals were divided into control (n = 12, saline injection) and Al_2_O_3_-NP (n = 12, Al_2_O_3_-NP injection at 20 mg/kg body weight) groups. Each treatment was administered daily via the tail vein for four consecutive days.

### Spatial navigation task

Twenty-four hours after the last Al_2_O_3_-NP injection was administered, the rats were subjected to spatial reference memory tests for five consecutive days.

The Morris water maze test was performed to assess spatial memory and learning ability. The test was performed following the method described by Morris (1984[33]) with minor modifications (Deguil et al., 2010[14]) . The water maze consisted of a circular water pool (100 cm, diameter; 60 cm, height) that was virtually divided into four equal quadrants. The pool was placed in a room that was decorated with many cues and filled with water (22 ± 2 °C) to a height of 30 cm. The test was conducted in two sessions: the hidden platform test (acquisition session), during which we evaluated the latency to reach a platform placed at the north-eastern side of the device; and the probe test (without platform). The test was initiated by placing a rat in the pool facing the pool wall and allowing the animal to swim freely during the first trial to the visible platform. The animal was then allowed to swim to the submerged platform, which was placed 1 cm under the water surface, in three other trials. For each trial, the rats were expected to find the platform for within a maximum of 60 s. If an animal failed to find the platform after 60 s, it was guided to it by the researcher. It was then allowed to stay on the platform for 10 s, after which it was removed from the pool before the next trial was started. Each rat was subjected to four trials per day for four consecutive days. The test was started at 09:00 AM each day. There was 10-min inter-trial interval between two consecutive trials. Daily escape latency for each rat was calculated as the average of the results from the four trials. Probe trial was performed on the fifth day. In this test, the platform was removed from the pool and the animals were allowed 60 s to swim freely. The swimming time in the quadrant where the platform was placed during the first four days (North-East) was recorded.

### Biochemical analyses

#### Tissue preparation 

After the last trial, the animals were sacrificed by decapitation. The brains were carefully excised on ice-cooled glass plates, immediately rinsed with physiological solution, dried with filter paper, and weighed. Hippocampi were immediately isolated, set in liquid nitrogen, and then stored at -80 °C until analysis. Each sample was homogenised in phosphate-buffered saline (pH 7.4). The homogenates were centrifuged at 600 g and then recentrifuged at 13 000 g for 20 min at 4 °C. The protein, malondialdehyde (MDA), and thiol group levels in the supernatants were assessed. In addition, the activities of catalase (CAT), superoxide dismutase (SOD), glutathione peroxidase (GPx), and acetylcholinesterase (AChE) were evaluated. 

#### Antioxidant enzyme assays

Total protein level in the hippocampus was determined as previously described (Hartree, 1972[21]) using bovine serum albumin as the standard. Lipoperoxidation was estimated spectrophotometrically at 532 nm by measuring brain MDA level (Ohkawa et al. 1979[36]). The results were expressed in nmoles of MDA/mg of protein. CAT activity was assayed by ultraviolet spectrophotometry at 240 nm (Aebi, 1984[3]), after which the results were expressed in µmoles H_2_O_2_/min/mg of protein. SOD activity was determined as previously described (Misra and Fridovic, 1972[30]) by spectrophotometry at 420 nm. The results of the analysis were expressed in U/min/mg of protein. GPx activity was measured by the method described by Flohé and Günzler (1984[17]), and expressed in U/mg/min. Tissue levels of sulfhydryl groups (expressed in Mm) were determined as described by Ellman (Ellman, 1959[15]).

#### AChE activity

AChE activity was assessed by the Ellman method (Ellman et al., 1961[16]). Changes in absorbance were measured for 15 min at 30-s intervals at 412 nm using a microplate reader. Results were expressed in µmoles of acetylthiocholine iodide hydrolysed/min/mg of protein (U AChE).

### Estimation of Al concentration 

Al concentration in the hippocampus was determined as follows. First, each sample was incinerated at 550 °C for 48 h in an oven muffle (Stuart, Staffordshire, UK) to obtain a white residue, which was then cooled to room temperature. Next, 1.25 ml of concentrated nitric acid was added to each residue for sample recovery. The volume of each mixture was then increased to 12.5 ml with ultra-pure water. Al concentration was measured by inductively coupled plasma-atomic emission spectroscopy.

### Fe, Ca, and Mg levels

Ionisable Ca and Mg levels were quantified using commercial kits (Biomaghreb, Ariana, Tunisia). The level of free Fe was determined by the ferrozine method (Leardi et al., 1998[26]) using a commercial kit (Biomaghreb). The ferrozine method is based on the following. At a pH of 4.8, Fe^3+^ is liberated from transferrin and reduced to Fe^2+ ^by ascorbic acid. A colourful complex that is spectrophotometrically measurable at 560 nm is formed from the reaction between ferrozine and the reduced Fe^2+^ ion. 

### Histological study

Immediately after decapitation, brains samples from each animal were fixed in 10 % formalin for 10 days. The fixed tissues were dehydrated in a graded series of ethanol and xylene solutions, and then embedded in paraffin. The brain samples were cut into 5-μm-thick sections using a microtome, deparaffinised, and rehydrated. Next, the sections were stained with haematoxylin and eosin (H&E), washed, and dried. Slides of the samples were prepared and viewed under a light microscope. Photomicrographs were taken for analysis.

### Statistical analysis

Results have been presented as mean ± standard error of the mean. Behavioural data were analysed using two-way analysis of variance, with Al_2_O_3_-NP treatment and repeated measures as principal factors. In the case of significant interaction (p ≤ 0.05), post hoc Fisher's least significant difference analysis was used to compare the control and treated groups. Oxidative stress data were analysed using *t*-test.

## Results

### Characterization of aluminum oxide nanoparticles

#### TEM analysis

**Figure 1:**
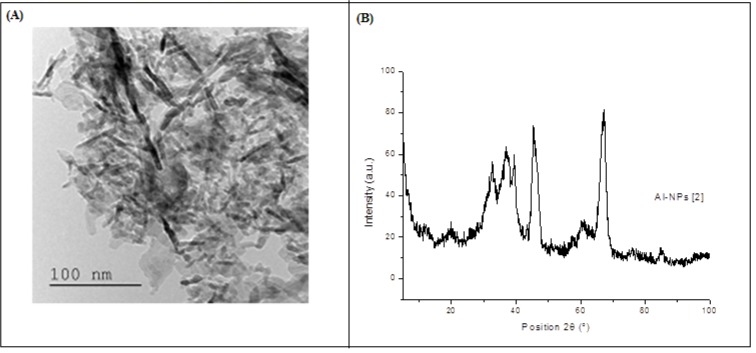
(A) Transmission electron microscopy (TEM) image of aluminium oxide nanoparticles. (B) Characterisation of aluminium oxide nanoparticles (Al_2_O_3_-NPs) by X-ray diffraction analysis (patterns for the crystal quality of Al_2_O_3_-NPs).

The TEM measurements (Figure 1A(Fig. 1)) have shown very thin Al_2_O_3_ particles (nanopowder, < 50 nm). 

#### Powdered X-ray diffraction (XRD) analysis

The XRD results (Figure 1B(Fig. 1)) showed five dominant peaks [36.53 u, 37.72 u, 39.46 u, 47.80 u and 67.01 u], which confirm the crystalline nature of the Al_2_O_3_-NPs. The same peaks were obtained by Pakrashi et al. (2013[37]).

### Spatial memory

**Figure 2:**
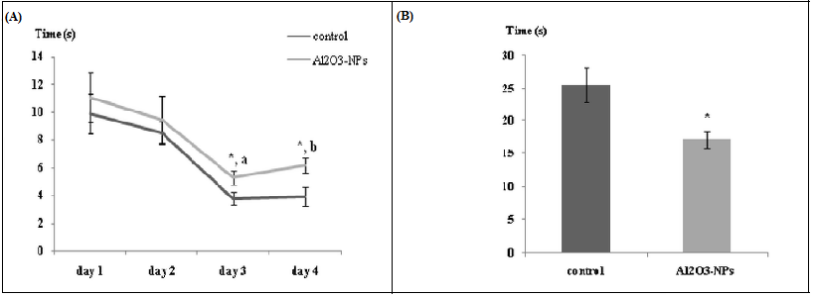
(A) Latency to find platform in the Morris water maze test (n = 6). Data are averaged by day and are presented as mean ± standard error of the mean. (a) Significant diminution in the latency to find the platform between days 1 and 3. (b) Significant training effect (p ≤ 0.00001). * indicates significant difference in the effect of the aluminium oxide nanoparticles (p ≤ 0.05) on the same day of training. (B) Time spent in the target quadrant in the Morris water maze test (Probe test). Data are presented as mean ± standard error of the mean. * indicates significant difference in the effect of the aluminium oxide nanoparticles (p ≤ 0.05).

The results obtained from the Morris water maze test are presented in Figure 2A(Fig. 2). A significant decrease in the latency to find the platform (F(3,30) = 15.17, p ≤ 0.00001) was observed throughout the four days in each group. Additionally, the time required to get to the platform was influenced by treatment on the third and fourth days (F(1,10) = 5.30 (p ≤ 0.05) and F(1,10) = 6.66 (p ≤ 0.05), respectively). The latency to find the platform decreased over the course of acquisition training (interaction treatment X repeated measure F(3,30) = 0.139, p ≥ 0.05). During the probe test (Figure 2B(Fig. 2)), NP-treated animals spent significantly less time (28.72 %) in the target quadrant (north-eastern side) than the control animals did (42.61 %) (F(1,10) = 8.54, p ≤ 0.05).

### Oxidative stress

**Figure 3:**
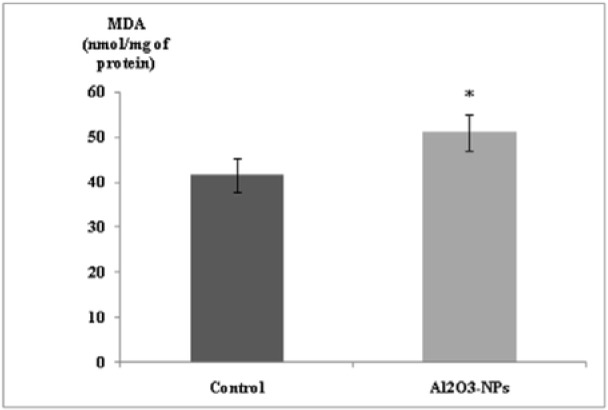
Effect of aluminium oxide nanoparticles (Al_2_O_3_-NPs) on malondialdehyde level in the hippocampus (n = 6). Data are presented as mean ± standard error of the mean. * indicates significant difference in the effect of the Al_2_O_3_-NPs (p ≤ 0.05).

Our findings revealed that MDA levels significantly increased in the hippocampal tissues of the treated rats (Figure 3(Fig. 3)). In addition, the Al_2_O_3_-NP formulation significantly inhibited SOD activity (p ≤ 0.05); however, it did not change thiol group levels or the activities of CAT and GPx (Table 1(Tab. 1)).

### AChE activity

**Figure 4:**
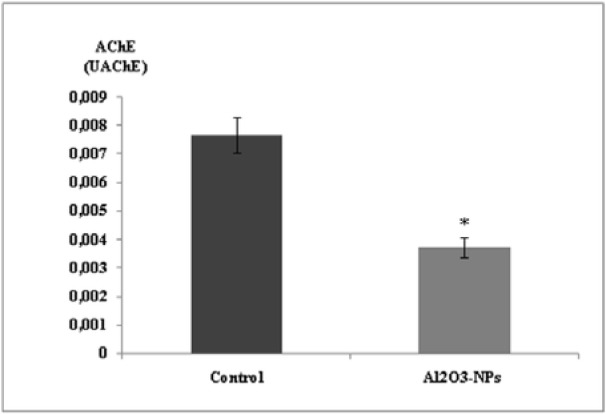
Effect of aluminium oxide nanoparticles (Al_2_O_3_-NPs) on acetylcholinesterase activity in the hippocampus (n = 6). Data are presented as mean ± standard error of the mean. * indicates significant difference in the effect of the Al_2_O_3_-NPs (p ≤ 0.05).

Our findings showed that the Al_2_O_3_-NPs significantly inhibited AChE activity in the hippocampus (Figure 4(Fig. 4)).

### Al level and mineral content

Al levels in the control and Al_2_O_3_-NP-treated groups are shown in Table 2(Tab. 2). Sub-acute Al_2_O_3_-NP treatment caused a significant increase in Al concentration in the hippocampus (p ≤ 0.05), which disturbed mineral balance in the hippocampus. Our data showed that Mg levels (p ≤ 0.05) were higher in the Al_2_O_3_-NP-treated group than they were in the control group. However, Ca and Fe levels significantly deceased in the hippocampi of Al_2_O_3_-NP-treated rats.

### Histological study

**Figure 5:**
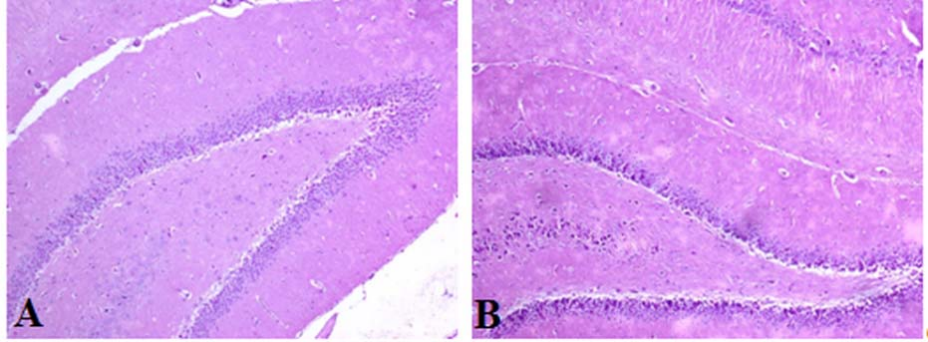
Photomicrographs of the rat hippocampus (H&E staining). No differences were observed when the hippocampi of the control (A) and treated rats (B) were compared (magnification=100X).

No morphological change was observed in the hippocampus of any rat in either group (Figure 5(Fig. 5)).

For additional results see the attached Supplementary data.

## Discussion

The purpose of this study was to examine the effects of Al_2_O_3_-NPs on spatial learning, memory, oxidative response, and bio-distribution of Al and other minerals in the rat hippocampus. The spatial learning capacities of rats were evaluated in the Morris water maze test. Our findings indicated that sub-acute Al_2_O_3_-NP treatment (20 mg/kg body weight) decreased the time needed to reach the platform with training in both groups, suggesting that rats in both groups learned to find the platform. Nevertheless, the Al_2_O_3_-NP-treated rats had a lower spatial performance than the control rats did, which was revealed by the probe test results. Additionally, the rats did not learn to accurately locate the escape point in the maze. Our findings are consistent with those reported by Kumar et al. (2011[25]) and Wang et al. (2014[52]), who administered oral (6-week treatment) and intraperitoneal injections (2-month treatment) of Al, respectively, to rats in their studies. Similar results were also obtained after intracerebral administration of Al to rabbits (Rabe et al., 1982[41]). The aforementioned effect of Al in the various laboratory animals could be attributed to interference with the activities of molecules that are implicated in long-term potentiation (Canales et al., 2001[10]), which results in learning deficits. 

The hippocampus is the site in the brain responsible for memory and learning. Particularly, the CA1 and CA3 areas are reported to be associated with long-term and short-term memory consolidation. 

In the present study, Al-NPs were injected into systemic pathways. Our findings showed an increased Al concentration in the hippocampi of the rats. Some authors have indicated that Al_2_O_3_-NPs can cross the BBB regardless of the route of administration (Shah et al., 2015[45]); however, there is insufficient evidence to confirm that. We believe that NPs are absorbed in the brain in their ionic form. Al is a neurotoxin that is implicated in some neurodegenerative diseases such as Alzheimer's disease (Abdel-Aal et al., 2011[1]; Gauthier et al., 2000[19]), in which patients suffer deterioration of some abilities such as attention, concentration, visual memory, vocabulary scores, and frontal lobe function (Kumar et al., 2011[25]).

Accumulation of Al in the hippocampus can partially contribute to the toxicity of Al_2_O_3_-NPs. This was confirmed in the present study by the disruption of spatial learning performance in the Al_2_O_3_-NP-treated rats. Many neurotransmitters such as acetylcholine (ACh) are involved in learning and memory processes. AChE activity is a good indicator of cholinergic system function (Zatta et al., 2002[55]). In the present study, significant inhibition of AChE activity was observed in the hippocampal homogenates of Al-NP-treated rats. The same results were reported by Kaizer et al. (2008[22]) The authors indicated that, after long-term oral administration of Al_2_Cl_3_ to mice, AChE activity decreased in homogenates of the cerebellum, hippocampus, and cerebral cortex of the animals. Furthermore, Kumar et al. (2009[24]) reported that Al can damage neurons and lead to depletion in AChE level. Moreover, Al alters the BBB and produces changes in noradrenergic and cholinergic neurotransmissions (Yokel, 2000[54]). Al and Al_2_O_3_-NPs can alter ion homoeostasis, including Ca^2+ ^homoeostasis, and decrease the release of ACh, which results in decreased AChE levels. This was confirmed in the present study by the significant decrease in Ca^2+^ levels in the hippocampi of rats treated with Al_2_O_3_-NPs. It has been suggested that Al interferes with the neurotransmission of glutamate and impairs hippocampal long-term potentiation. 

Oxidative stress is the most common mechanism through which toxicity occurs following exposure to NPs (Yang et al., 2009[53]). Our results showed that, MDA levels increased significantly while SOD activity decreased in the hippocampi of the Al_2_O_3_-NP-treated rats. Sethi et al. (2008[44]) reported similar findings after administering oral Al to rats. Inhibition of SOD activity leads in part to increase in lipid peroxidation (Kumar et al., 2009[24]). Furthermore, it disturbs Fe homoeostasis, which results in excessive levels of free Fe ions, which causes oxidative damage by the Fenton reaction and further leads to neurodegeneration. No differences were observed between the two groups with regards to thiol group levels and the activities of CAT and GPx. 

In addition, our results showed that Al_2_O_3_-NPs can disrupt the metabolism of mineral elements that are necessary for antioxidant enzyme synthesis in the rat brain. Flora et al. (2008[18]) reported that the production of reactive oxygen species is related to decrease in the levels of some antioxidant enzyme cofactors such as Fe, Zn, Mg, and Cu. In the present work, the most pronounced oxidative damage was observed as a result of an excessive MDA level in the hippocampus. Moreover, the Al_2_O_3_-NPs might have induced free radical generation that further initiated lipid peroxidation and damaged cellular components. Previous studies have shown that Al can induce lipid peroxidation in the brain and cause neurodegeneration (Kumar et al., 2009[24]; Tripathi et al., 2009[49]), which can be confirmed by increased MDA levels and inhibition of SOD activity in the brain (Morsy et al., 2013[34]). Furthermore, Al can cause neuronal inflammation, which leads to a decline in visoperception and attention, and impairment in working and semantic memories (Platt et al., 2001[39]). Our data revealed no difference in hippocampus structure between the control and treated groups.

Al-NPs can alter the neural membrane by reducing its lipoprotein integrity (Banks et al., 2006[8]). This induces partial damage to the BBB, which leads to Al accumulation in the brain. It has been reported by Rebai and Djebli (2008) that increase in Al levels in the hippocampus and cortex causes damages, such as neurofibrillary degeneration, to these regions. Many studies have attributed NP-induced toxicities to oxidative stress and inflammatory reactions (Adamcakova-Dodd et al., 2014[2]; Ma et al., 2010[29]).

In conclusion, our findings suggest that short-term systemic exposure to Al_2_O_3_-NPs induces oxidative stress in the hippocampus. We also found that the latter possibly results from Al accumulation in the hippocampus and leads to changes in metabolic activity, thereby affecting learning and memory in rats.

## Funding sources

This research did not receive any specific grant from funding agencies in the public, commercial, or not-for-profit sectors.

## Conflicts of interest

None.

## Supplementary Material

Supplementary data

## Figures and Tables

**Table 1:**
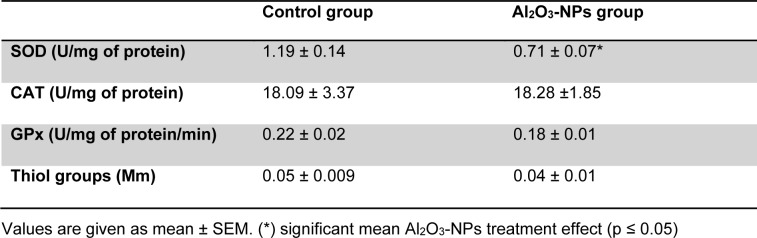
Effect of four Al_2_O_3_-NP injections on CAT, SOD and GPx activities in hippocampus (n = 6)

**Table 2:**
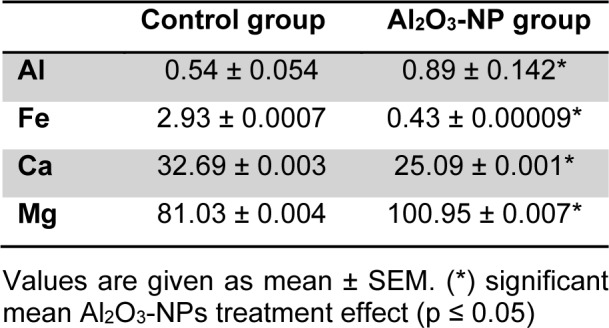
Al (n = 3) and mineral content (n = 5) in hippocampus of rats after four Al_2_O_3_-NP injections compared to control. Values are expressed in µg/g fresh weight.
