# Supplementary material for: Aluminium oxide nanoparticles compromise spatial learning and memory performance in rats
Source: EXCLI J. 2018 Feb 14;17:200–10. doi: 10.17179/excli2017-1050 (PMC5938538; doi:10.17179/excli2017-1050)
Supplement: Supplementary data [file EXCLI-17-200-s-001.pdf]

EXCLI Journal 2018;17:200-210 – ISSN 1611-2156
Received: December 20, 2017, accepted: February 10, 2018, published: February 14, 2018

Supplementary data to:

Original article:

ALUMINIUM OXIDE NANOPARTICLES COMPROMISE SPATIAL LEARNING AND MEMORY PERFORMANCE IN RATS

Imen M'rad[1*], Mustapha Jeljeli[2], Naima Rihane[1], Pascal Hilber[3], Mohsen Sakly[1], Salem Amara[4]

[1] Laboratoire de Physiologie Intégrée, Faculté des Sciences de Bizerte, Université de Carthage, Tunisie
[2] Institut Supérieur des Sciences Humaines de Tunis, Université El Manar, Tunisie
[3] Centre de recherche sur les fonctionnements et dysfonctionnements psychologiques CRFDP EA 7475, Université de Rouen Normandie, France
[4] College of Education Afif, Ministry of Education Shaqra University , Kingdom of Saudi Arabia

[*] Corresponding author: Imen M'rad, Faculty of Science of Bizerte, Laboratory of Integrated Physiology, Jarzouna 7021, Carthage University, Tunisia. Telephone: 0021650481303; Fax: 0021672590566; E-mail: mrad.amen@gmail.com

http://dx.doi.org/10.17179/excli2017-1050

This is an Open Access article distributed under the terms of the Creative Commons Attribution License (http://creativecommons.org/licenses/by/4.0/).

EXCLI Journal 2018;17:200-210 – ISSN 1611-2156
Received: December 20, 2017, accepted: February 10, 2018, published: February 14, 2018

Table 1: Reference Memory: Raw data in Morris water maze test

Groups	Day 1	Day 2	Day 3	Day 4	Day 5 (Probe test)
**T1**	15,75	6,25	5,25	2,75	21,5
**T2**	6,5	8	4	3,25	21
**T3**	6,5	9,75	3,75	4,5	20
**T4**	11,5	6,5	4,25	5,5	28
**T5**	9	9	2,25	4,25	36,8
**T6**	10	11,5	3,25	3,25	26,1
**Average**	9,875	8,5	3,79166667	3,91666667	25,5666667
**SEM**	1,4226589	0,819044158	0,41036908	0,41666667	2,58697593
**Al1**	19,5	11	7,25	7	12,3
**Al2**	8,75	17	4,5	5,75	20
**Al3**	7,75	6	4,75	6,75	20,1
**Al4**	8	7	5,25	6,5	18
**Al5**	10,75	6,5	4,75	3,75	17,3
**Al6**	11,75	9,25	4,75	7,25	15,7
**Average**	11,0833333	9,458333333	5,20833333	6,16666667	17,2333333
**SEM**	1,80239039	1,693635964	0,42039994	0,52704628	1,19935168

- T1, T2, T3, T4, T5 and T6 refer to control rats
- Al1, Al2, Al3, Al4, Al5 and Al6 refer to Al_2O_3 nanoparticles treated rats
- Day 1, day 2, day 3, day 4: refer to the latency (per second) to reach the platform refuge in the water maze during the 4 training days
- Day 5: refers to the time spent (in second) by each rat in the target quadrant (North-East)

EXCLI Journal 2018;17:200-210 – ISSN 1611-2156
Received: December 20, 2017, accepted: February 10, 2018, published: February 14, 2018

Table 2: Oxidative stress indicators and acetylcholinesterase activity (n=6)

Groups	MDA (nmol/mg protein)	SOD (U SOD/min/mg protein)	CAT (µmole H_2O_2/min/mg protein)	GPx (U/mg protein/min)	Thiol (Mm)	AChE (UAChE)
**T1**	45,8511891	1,578868124	16,6658302	0,34564182	0,04553	0,00949785
**T2**	46,196689	1,331595656	12,4939839	0,17202453	0,08478	0,00731504
**T3**	25,2355193	0,640643087	9,49100869	0,17960151	0,03768	0,00687069
**T4**	35,3126951	1,415475697	33,2025164	0,21528923	0,07693	0,00692464
**T5**	47,0773299	1,298614958	16,7537362	0,22065493	0,07065	0,008999
**T6**	49,2729922	0,899503386	19,9889641	0,24042193	0,02355	0,00633494
Average	41,4910691	1,194116818	18,0993399	0,22893899	0,05652	0,00765205
SEM	3,8093453	0,143757186	3,37177302	0,02561236	0,009962595	0,00062318
**Al1**	50,7043338	0,718650996	11,8000719	0,13994631	0,02041	0,00279915
**Al2**	43,3549074	0,819075026	24,2688897	0,20328731	0,02512	0,00248182
**Al3**	55,9006263	0,818234086	15,1524831	0,17594664	0,0471	0,00443666
**Al4**	43,4337607	0,845132556	20,3457837	0,21145298	0,03925	0,00466393
**Al5**	66,5025694	0,364908425	21,2863248	0,18788091	0,08949	0,00454963
**Al6**	47,9204454	0,701425805	16,8861768	0,17761184	0,07693	0,0039848
Average	51,3027738	0,711237816	18,289955	0,18268766	0,049716667	0,00381933
SEM	3,60121395	0,073280317	1,85189299	0,01029043	0,011405786	0,00035797

- T1, T2, T3, T4, T5 and T6 refer to control rats
- Al1, Al2, Al3, Al4, Al5 and Al6 refer to Al_2O_3 nanoparticles treated rats
- MDA (nmol/mg protein): Malondialdehyde levels in hippocampi expressed in nmol per mg of protein in hippocampus
- SOD (U SOD/min/mg protein): superoxide activity in hippocampus was expressed in arbitrary unit per minute per mg of protein in hippocapus
- CAT (µmole H_2O_2/min/mg protein): Catalase activity expressed in µmoll of H_2O_2 per minute per mg of protein in hippocampus
- GPx (U/mg protein/min): glutathione peroxidase activity in hippocampus expressed in arbitrary unit per mg of protein in the same structure per minute
- Thiol (Mm): Thiol groups levels are expressed per Mm
- AChE (UAChE): acetylcholinesterase activity expressed by arbitrary unit of acetylcholinesterase

EXCLI Journal 2018;17:200-210 – ISSN 1611-2156
Received: December 20, 2017, accepted: February 10, 2018, published: February 14, 2018

Table 3: Fe, Ca, Mg and Al content of hippocampus (n=3-5)

Groups	Fe level .10^{-3} mg/mg fresh weight	Ca level .10^{-3} mg/mg fresh weight	Mg level .10^{-3} mg/mg fresh weight	Al level (mg/mg fresh weight)
**T1**	0,00186813	0,03749044	0,0952381	0,59124424
**T2**	0,00120879	0,03978577	0,07368421	0,44009662
**T3**	0,0043956	0,02448355	0,07046632	0,61116279
**T4**	0,00428571	0,02754399	0,07564767	
**T5**	0,00444444	0,03350785	0,09015544	
**Average**	0,00293956	0,03269232	0,08103835	0,54750122
**SEM**	0,00073374	0,00304313	0,00489727	0,05400925
**Al1**	0,00021978	0,02371844	0,0952381	1,16694215
**Al2**	0,00032967	0,0283091	0,1047619	0,69292929
**Al3**	0,00076923	0,02524866	0,1047619	0,81215278
**Al4**	0,00032967	0,02218822	0,07619048	
**Al5**	0,00054945	0,02601377	0,12380952	
**Average**	0,00043956	0,02509564	0,10095238	0,89067474
**SEM**	9,8289E-05	0,00103785	0,00773718	0,14235674

- T1, T2, T3, T4 and T5 refer to control rats
- Al1, Al2, Al3, Al4 and Al5 refer to Al_2O_3 nanoparticles treated rats
- Fe level: iron level in one mg fresh tissue of hippocampus
- Ca level: calcium level in one mg fresh tissue of hippocampus
- Mg level: magnesium level in one mg fresh tissue of hippocampus
- Al level: aluminium level in one mg fresh tissue of hippocampus
